# Metadata harmonization from biological datasets with language models

**DOI:** 10.1093/bioadv/vbaf241

**Published:** 2025-10-01

**Authors:** Alexander Verbitsky, Patrick Boutet, Mohammed Eslami

**Affiliations:** Netrias, LLC, Annapolis, MD 21401, United States; Netrias, LLC, Annapolis, MD 21401, United States; Netrias, LLC, Annapolis, MD 21401, United States

## Abstract

**Motivation:**

Integrating biomedical datasets is hindered by inconsistent metadata, where the same concept may be represented in many ways (e.g. “Ca.,” “Carcinoma,” “tumor” for “Neoplasm”). Metadata harmonization automatically converts these researcher-specific terms into standard vocabulary terms to enable downstream integration. Current solutions, such as Common Data Elements and laboratory information management systems, either require navigating thousands of subtly different terms or disrupt researcher workflows, resulting in siloed datasets. This fragmentation forces researchers to spend over 40% of curation time on manual standardization.

**Results:**

We present a language model-based harmonization solution that automatically maps researcher-specific metadata to standard terms across domains including cancer, alcohol research, and infectious disease. Our method fine-tunes GPT-2 models with realistic data augmentation, generating term variations that mimic researchers’ documentations, such as typos, abbreviations, and word reordering. This enables harmonization even in domains without curated synonym sets. Fine-tuned models achieve 96% in-dictionary accuracy, reducing manual effort by over 90% when the term exists in the vocabulary, and 17% out-of-dictionary accuracy for previously unseen standards, outperforming traditional heuristics and zero-shot GPT-4o. Larger general models provide modest gains for unseen terms, while domain-specific small models achieve superior performance on specialized terminology, delivering a scalable, low-burden solution for harmonizing biomedical metadata and accelerating downstream data integration.

**Availability and implementation:**

All datasets used in this study, including training, validation, and test splits, are available via the Netrias Hugging Face organization. This includes datasets for the cancer and alcohol-bacteria mix domains used to develop and evaluate harmonization models. Experiment results are provided in the Supplementary Materials. We also share one representative GPT-2 Large cancer model and five GPT-2 Large models trained on different alcohol-bacteria domain mixtures: (100/0, 75/25, 50/50, 25/75, 0/100). All resources are released under the Apache 2.0 license to support reproducibility and reuse.

## 1 Introduction

Biomedical research generates vast amounts of data, but inconsistent metadata—the annotations researchers provide to describe samples, assays, or conditions—makes these datasets difficult to integrate (Liang *et al.* 2022). Inconsistencies mainly arise because data are generated across sources and modalities (e.g. genomics, transcriptomics, proteomics), institutions, and individual researchers, leading to diverse representations of the same concept. For example, the standard “Lung Cancer” may appear as “Lung Ca,” “Cancer of Lung,” or “LC” in different datasets. Converting these variations into a single standard term from a controlled vocabulary is known as metadata harmonization. When metadata are not harmonized, datasets remain fragmented and siloed, and analysts spend over 40% of curation time on manual standardization (Gomez-Cabrero *et al.* 2014, Bucher *et al.* 2019, Gonçalves and Musen 2019).

Several standardization resources exist to promote consistency, such as ontologies, controlled vocabularies, and Common Data Elements (CDEs). Biomedical research offers many examples: synthetic biology standards (McLaughlin *et al.* 2020, Schoch *et al.* 2020), the National Cancer Institute Thesaurus (NCIt) for cancer (Metadata Services for Cancer Research (CBIIT), Schriml *et al.* 2019), the Infectious Disease Ontology (He *et al.* 2020, Babcock *et al.* 2021), and clinical trial templates (Chan *et al.* 2013, Kim *et al.* 2019, Lin *et al.* 2020). However, these resources are rarely used consistently, as researchers often rely on lab-specific, ad hoc naming conventions, and data submitted to public repositories are frequently disconnected from formal standards (Sansone *et al.* 2019, Liang *et al.* 2022).

Current technologies to enforce standardization, such as electronic lab notebooks (Schröder *et al.* 2022) and web-based data entry forms (Gonçalves *et al.* 2017, Morrell *et al.* 2017), attempt to bring the ontology to the researcher, but they disrupt workflows by requiring users to navigate thousands of terms or select from long drop-down lists before even running an experiment. Existing natural language processing and machine learning approaches are also limited because they require long text passages (e.g. publication abstracts) rather than the short, context-poor metadata entries found in spreadsheets or file names (Kersloot *et al.* 2020, Steppi *et al.* 2020, Hoyt *et al.* 2022).

To address these challenges, we present a language model-based approach for metadata harmonization that works with minimal context. Our method automatically standardizes researcher-specific terms to a controlled vocabulary after observing a single variant, reducing the manual curation burden. We first demonstrate the approach in cancer research, a domain with well-curated vocabularies and known term variations, where fine-tuned language models achieve over 96% in-dictionary accuracy, meaning they reliably harmonize new variants of known vocabulary terms, dramatically reducing manual effort. We then extend the approach to alcohol research, where synonym coverage is narrow, using realistic data augmentation to train models. Finally, we evaluate how domain-specific models compare to larger general models like GPT-4o, showing that small, fine-tuned models outperform general ones on specialized terminology, while larger models provide modest advantages for previously unseen, out-of-dictionary terms.

## 2 Methods

### 2.1 Data sources and preparation

Ontologies for cancer, alcohol, and bacteria terminology were constructed from authoritative sources, each undergoing domain-specific processing. Cancer vocabulary terms were exported from NCIt, caDSR, GDC, ICD-O3, and MedDRA, and semantic type information from NCIt was extended to all standard items by matching texts to terms with existing semantic types. The alcohol vocabulary combined terms from PhenX Toolkit, WHO Lexicon of Alcohol and Drug Terms, and HRB National Drugs Library, with a held-out annotated dataset from WCAAR reserved for evaluation. Bacteria terms were drawn from NCBI Taxonomy and supplemented with NIST conventions, merging scientific names while filtering to bacteria-only entries.

Common processing steps were applied across all domains for data consistency and quality. Each source was normalized to standard-synonym pairs, with each standard term also appearing as its own synonym. Redundant entries were removed by collapsing duplicates after case normalization and whitespace trimming, ensuring that each unique synonym-standard pair appeared only once. Multimappings, where a synonym mapped to multiple standards, were identified using case-insensitive comparison, stored separately, and removed from the primary dataset to prevent ambiguous training signals. For remaining ambiguities, we selected the candidate with the highest string similarity to the synonym, preferring title case text to maintain a learnable, syntactically representative mapping.

The fine-tuning datasets were built from these cleaned standard-synonym pairs. For cancer, the training set included 35 828 standards with an average of two variations per standard (range 1–59). Validation and test sets were small, each containing one variant per standard, and were randomly sampled from the remaining standards to provide unbiased evaluation. For alcohol-bacteria mixtures, each training set contained ∼26 600 standards, where synthetic augmentation created substantially more variants per standard. Depending on the domain mix, the average variations ranged from 8 (100% bacteria) to 12 (100% alcohol), with maxima exceeding 2000 variants per standard in bacteria-heavy datasets.

All training, validation, and test splits are available via the Netrias Hugging Face organization.

### 2.2 Harmonization heuristics

Exploratory analysis applied exact, case-insensitive, and similarity-based heuristic matching to suggest potential standards. Exact matches were used when the input text perfectly aligned with a known standard or synonym. Case-insensitive matching provided flexibility when the input text differed by capitalization. Additionally, a normalized indel similarity assessed how closely input terms matched existing standards. The indel distance calculates the minimum number of insertions and deletions required to change one sequence into another. This measure, calculated as 1 − (distance/(len_1_ + len_2_)), produces a score between 0 and 1. A threshold of 0.8 was applied to identify matches, accounting for minor variations that exact matching does not handle.

### 2.3 Data augmentation

A multilevel data augmentation approach was implemented to generate realistic term variations for ontologies lacking diverse synonyms. This approach operates at four levels: character, sub-word, word, and structural, applying functions to create synthetic synonyms that mimic real-world variations.

Character-level modifications simulate typographical errors and formatting changes through operations such as duplication, deletion, insertion, and transposition of characters, including keyboard proximity-based substitutions. Sub-word manipulations modify parts of words by removing common endings, replacing phonemes, and altering punctuation. Word-level techniques generate concise representations by removing non-essential words, replacing terms with synonyms, and creating abbreviations. Structural-level augmentations modify the organization of multiword terms by altering numerical and textual element positions, manipulating grouping symbols, and rearranging words around conjunctions or delimiters.

The augmentation process involves selecting a subset of operations most relevant to the target domain from the full suite of functions. For example, in the case of the alcohol vocabulary, eight specific operations were chosen, including manipulations of number-text positions, grouping symbols (both removal and content manipulation), conjunctions, delimiters, keywords (replacement and removal), and stop words. These selected operations are then applied to existing terms, ensuring generated synonyms realistically represent expected variations while exploding the vocabulary. This approach creates a richer, more diverse model training dataset, addressing initial data scarcity.

### 2.4 Model architectures

LLMs and small NMT models were used for the harmonization task. Four GPT-2 variants—base (124M parameters), Medium (355M), Large (774M), and XL (1.5B)—were fine-tuned using the Hugging Face Transformers library. Two character-level NMT models, a BiLSTM network (7M) and a Transformer (550K), were implemented and trained using Keras.

The NMT models were designed to process input sequences at the character level. The BiLSTM model has an encoder-decoder architecture. The encoder comprises an embedding layer followed by a bidirectional LSTM layer, while the decoder includes an embedding layer, an LSTM layer, and a dense output layer. The encoder’s final hidden states initialize the decoder’s state, allowing the model to capture contextual information from both past and future tokens in the input sequence. The character-based Transformer adapts the standard Transformer architecture for character-level processing. It incorporates positional embeddings to encode sequence order, multihead attention mechanisms to capture character relationships, and feed-forward neural networks in both encoder and decoder. Layer normalization and residual connections throughout the structure stabilize training and facilitate gradient flow.

### 2.5 Model training and evaluation

LLMs and NMT models followed different training approaches due to their architectural differences. For LLMs, input data was formatted with the prompt The standardized form of “synonym” is “standard,” while NMT models were trained to directly output the harmonized form given an input term without a specific prompt structure. The cancer dataset included 141K entries, and the alcohol dataset contained 200K. The validation datasets had 200 items, while the ID and OOD test sets had 100 each.

Hyperparameter optimization used a combination of manual tuning and grid search. For LLMs, key hyperparameters included a maximum of 100 training epochs, per-device train and evaluation batch sizes of 8, and the AdamW optimizer with a learning rate scheduler employing a linear warmup followed by a linear decay. Weight decay was set to 0 to prevent over-regularization. For NMT models, hyperparameters included a maximum of 300 epochs, a batch size of 256, a latent dimensionality of 512 for the encoding space, and an embedding dimension of 64. The Transformer-based NMT model used eight attention heads and one encoder/decoder layer. The BiLSTM model used the RMSprop optimizer with a learning rate of 0.001, while the Transformer model used the Adam optimizer with a Noam learning rate schedule. Both NMT models used sparse categorical cross-entropy as the loss function.

Model robustness was enhanced through dropout and a callback system implementing early stopping techniques. LLMs were evaluated every 10% of an epoch using a beam search with five beams, five return sequences, and a diversity penalty of 0.8 to encourage varied outputs. These models used a patience of 20 evaluations, monitoring validation ID accuracy for early stopping, and included callbacks for saving tokenizers alongside model checkpoints. NMT models, evaluated at the end of each epoch, employed an early stopping callback monitoring validation loss with a patience of 10 epochs and a minimum delta of 0.0001. Additional callbacks for NMT models included checkpointing and detection of NaN losses to identify potential training instability. For all architectures, the best-performing model based on their respective monitored metrics was automatically loaded at the conclusion of training.

For the LLM models, outputs were generated using beam search with a width of five, producing the top candidate harmonizations. These candidates were then refined by cross-referencing with the multimappings identified during data preparation. If a match was found, all related mappings were included in the final top outputs, enriching the model’s predictions with potential alternative standardizations. Beam search was not used for the NMT models.

Model performance was evaluated using a combination of accuracy and additional metrics. ID accuracy evaluated the model’s ability to harmonize new synonyms for known standards, while OOD accuracy assessed generalization to unseen standards and synonyms. Beyond accuracy, additional metrics included BiLingual Evaluation Understudy (BLEU) score and Character Error Rate (CER). BLEU score quantifies n-gram precision between model outputs and reference standards, offering a similarity measure from 0 (no overlap) to 1 (perfect match). CER, calculated as the Levenshtein distance divided by the standard term length, measured string-level differences between model predictions and correct terms.

For benchmarking, OpenAI’s GPT-4o model (gpt-4o-2024–08-06) was evaluated zero-shot across six datasets, with five iterations per test and batches containing up to 100 terms. The following system prompt was used to guide the model:

*You are tasked with harmonizing cancer-related terms to their corresponding standards, using your knowledge of cancer terminology. These terms span semantic types “Finding,” “Neoplastic Process,” “Disease or Syndrome,” “Laboratory Procedure,” and “Quantitative Concept.” Most standards come from the NCI Thesaurus.*

*Prefer standards that are written out in full (i.e. no abbreviations or acronyms), unless the context absolutely requires an abbreviation.*

*For each term provided, return:*

1. *The corresponding standard (i.e. the term that most closely matches the meaning and context of the input).*
2. *The top five possible standards, ranked from most to least likely.*

The user prompt then provided the model with a list of terms to harmonize:

*Please harmonize these terms: [list of terms]*

## 3 Results

### 3.1 Cancer vocabulary data collection

**Figure 1.**
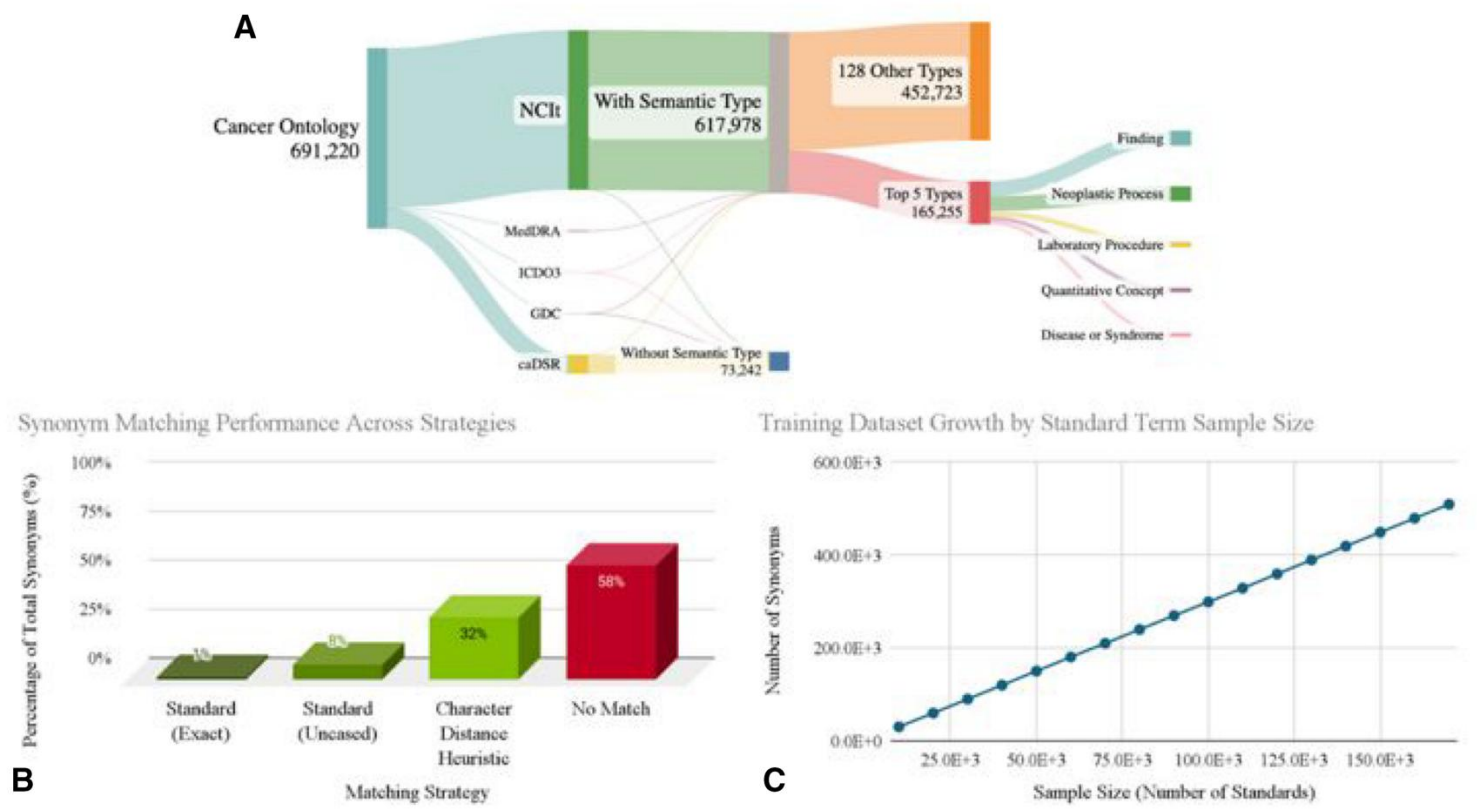
(A) Composition of the cancer dataset. Distribution of terms across categories, including those with semantic types (617 978), the subset of top five semantic types used for fine-tuning (165 255), and terms without semantic types (73 242). (B) Synonym matching performance across different strategies for 434 841 synonyms matched against 259 707 unique standards from our compiled cancer vocabulary. Standard (Exact): exact string matching; Standard (Uncased): case-insensitive matching; Character Distance Heuristic: fuzzy matching based on character differences; No Match: synonyms unmatched by any method. Fifty-eight percent of synonyms remained unmatched, demonstrating limitations of basic matching. (C) Fine-tuning dataset growth as a function of standard term sample size, showing a near-linear relationship between the number of standard terms included and the total number of synonyms in the dataset.

Cancer is one of the most studied domains in biomedical research and as a result has no shortage of data or ontologies. We took advantage of a training corpus that maps malformed researcher annotations from data, or synonyms, to the standard term (or command data element) within a controlled vocabulary. We combined terms from multiple sources, including NCIt (Golbeck *et al.* 2003), Cancer Data Standards Registry and Repository (caDSR) (Brady *et al.* 2024), Genomic Data Commons (GDC) (Grossman *et al.* 2016), International Classification of Diseases for Oncology (ICD-O3) [International Classification of Diseases for Oncology, 3rd Edition (ICD-O-3)], and Medical Dictionary for Regulatory Activities (MedDRA) (Brown *et al.* 1999). Our compiled training dataset contains a total of 691 220 terms (Fig. 1A). Most of these terms (617 978) are associated with semantic types, a NCIt classification system that categorizes terms based on their nature or role within the biomedical domain. We focused on the five largest semantic types: “Finding,” “Neoplastic Process,” “Disease or Syndrome,” “Laboratory Procedure,” and “Quantitative Concept.” This subset, comprising 165 255 terms, not only represents the most populous groups within the dataset, but also provides a more manageable scope for the harmonization task. Chemical names and procedural semantic types were excluded (specifically, “Pharmacologic Substance,” “Organic Chemical,” “Therapeutic or Preventive Procedure,” and “Intellectual Product”) to concentrate on terms that follow more typical natural language structures rather than technical jargon. Additionally, 154 432 ambiguous term mappings, cases where a single representation mapped to multiple standards, were identified. To avoid ill-formed training examples, we retained only one mapping per ambiguous synonym, selecting the closest match by string similarity.

With this dataset, we wanted to assess the scale of the harmonization problem. Namely, can simple methods, such as capitalizations, direct matches to previous synonyms, or string distances solve the standardization problem (Fig. 1B). In this context, a synonym is a malformed representation of a standard term that conveys the same or similar meaning. Synonyms can take various forms, including acronyms (e.g. “MRI” for “Magnetic Resonance Imaging”), abbreviations (e.g. “Ca.” for “Cancer”), lexical variants (e.g. “tumor” and “tumour”), word reorderings (e.g. “Cancer of the Lung” for “Lung Cancer”), or conceptually related terms (e.g. “Neoplasm” for “Tumor”). To simulate a real-world scenario where the association between synonyms and their standards is unknown, we tested all synonyms (434 841) against all standards (259 707) in our cancer vocabulary using various matching techniques. Standard exact matching, requiring perfect string alignment, successfully matches only a small fraction of terms. Even case-insensitive matching (Standard Uncased) yields only marginal improvement. A more sophisticated approach using an indel distance heuristic performs better, but still leaves a significant portion of terms unmatched. Fifty-eight percent of synonyms in our dataset remain unmatched using these conventional techniques. Moreover, heuristic approaches are limited to known standards, necessitating more advanced harmonization methods such as generative models which can potentially standardize unfamiliar terms.

To develop our AI-driven approach, we constructed a fine-tuning dataset consisting of 141 065 terms, including 83 904 synonyms of 57 161 standards. This dataset provides a diverse range of term variations and relationships for training our models. We observed a near-linear relationship between the number of standard terms and the total number of synonyms in our training dataset (Fig. 1C), indicating that, on average, each standard is associated with three synonyms. This results in a uniform density of term variations across the vocabulary. For evaluation, separate validation and test sets were created, each containing 200 terms. The validation set guided early stopping, tracked performance, and identified the best model during training. The test set was used to assess the final model’s performance.

### 3.2 Large language models for cancer metadata harmonization

**Figure 2.**
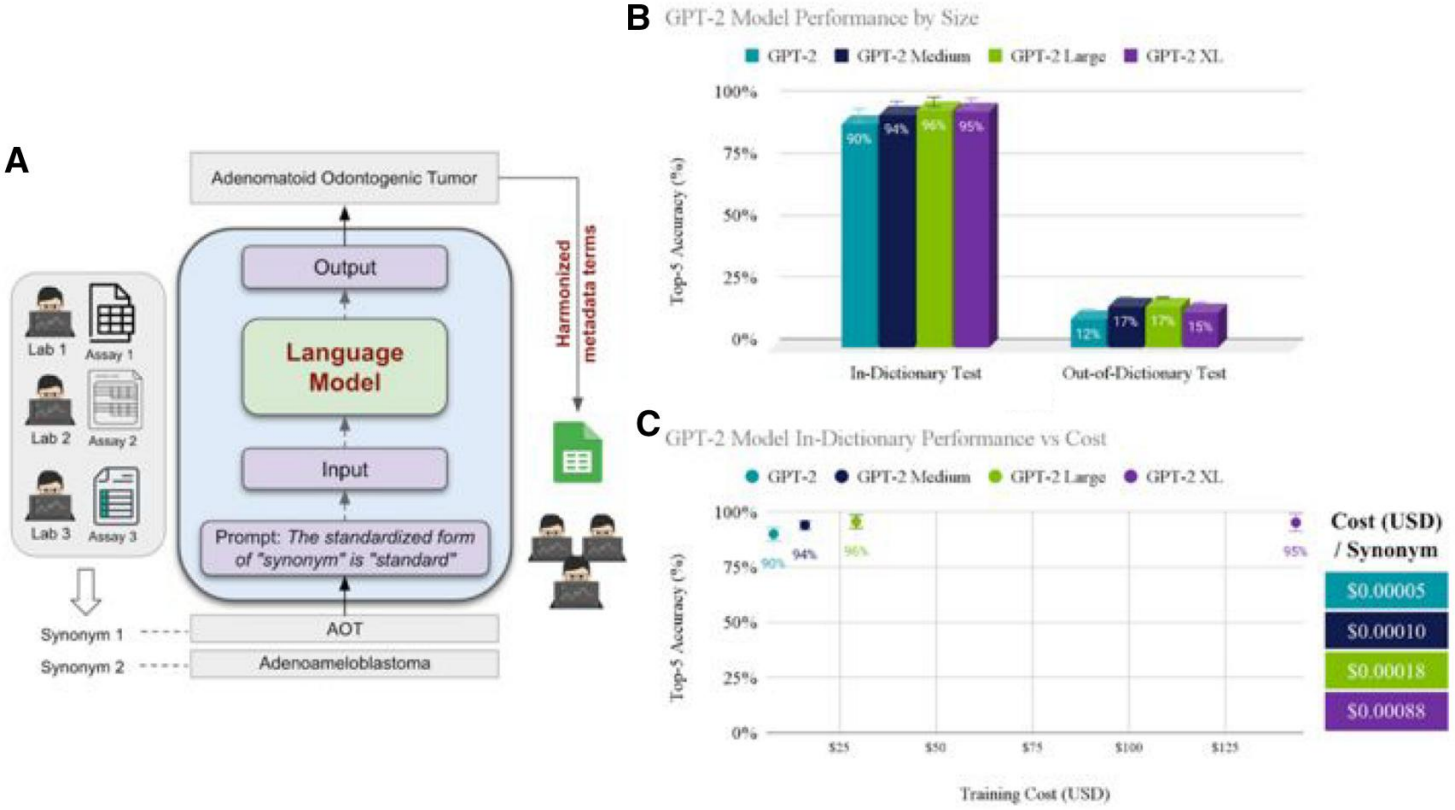
(A) The LLM-based harmonization system. The model takes a synonym as input, along with a prompt specifying the task, and outputs the standardized form of the term. (B) GPT-2 model top-5 performance by size for in-dictionary and out-of-dictionary cancer metadata harmonization tests. In-dictionary accuracy ranges from 90% to 96%, while out-of-dictionary accuracy is 12% to 17%. (C) GPT-2 model in-dictionary performance *versus* training cost. GPT-2 Large achieved 96% accuracy at $29, while GPT-2 XL reached 95% accuracy at $143.

Language models can map malformed representations of standards to their corresponding terms within a controlled vocabulary (Fig. 2A) (Qiang *et al.* 2023, Ronzano and Nanavati 2024, Li *et al.* 2025). Our focus here is to determine whether this mapping can occur with minimal context. Large language models (LLMs), trained on vast amounts of text, already capture much of the context a researcher might otherwise provide. LLMs can be used (i) fine-tuned on curated synonyms to learn direct mappings to standards (Li *et al.* 2025) or (ii) zero shot, where a prompt requests a harmonized output without task-specific training (Ronzano and Nanavati 2024). We evaluated both approaches.

LLMs were fine-tuned context-free on a curated cancer vocabulary dataset using the prompt The standardized form of “synonym” is “standard.” During fine-tuning, the model learns to predict the correct “standard” given the “synonym” and preceding text. This allows the model to learn the associations between synonyms and their standardized forms. During testing and application, the prompt was modified to “The standardized form of ‘synonym’ is,” creating a completion task. This approach challenges the model to generate the standardized term based on learned associations, applying the knowledge it acquired during fine-tuning. The resulting system automates harmonization while allowing researchers to continue using their preferred terminology, alleviating the burden of manual standardization.

GPT-2 was selected for this study due to its computational efficiency, well-documented fine-tuning process, and unrestricted commercial use. While newer, larger models could potentially offer better out-of-dictionary (OOD) performance, GPT-2 provides an efficient balance of performance and practicality. Five iterations of four different-sized GPT-2 model variants (GPT-2, GPT-2 Medium, GPT-2 Large, and GPT-2 XL) were fine-tuned and evaluated using in-dictionary (ID) and OOD accuracy metrics. All models were trained with an early stopping criteria, halting training after 20 consecutive evaluations without validation accuracy improvement at 0.05 epoch increments. Accuracy was measured with no credit given to partial matches, providing the most stringent assessment of model performance. ID accuracy evaluates the model’s ability to standardize new synonyms of standards present in the fine-tuning dataset, while OOD accuracy assesses its capacity to generalize to entirely new standards and their synonyms that the model has never seen before. The OOD test was made even more challenging by exclusively including terms from semantic types not encountered during training.

As expected, larger models generally performed better, with GPT-2 Large achieving the highest accuracies: 96% for ID and 17% for OOD tests (Fig. 2B). Interestingly, the largest model, GPT-2 XL, performed slightly worse than GPT-2 Large (95% ID, 15% OOD). The substantial gap between ID and OOD performance (78%–79% across models) underscores the challenge of generalizing to new concepts and semantic types in specialized domains. While models effectively standardize variations of familiar terms, they struggle with novel standards and synonyms, particularly from unseen semantic categories, highlighting the complexity of true out-of-domain generalization in biomedical nomenclature.

Given the requirement of fine-tuning, we also analysed the relationship between model performance and the cost of fine-tuning (Fig. 2C). GPT-2 Large achieved the highest ID accuracy at 96% with a training cost of $29, outperforming GPT-2 XL, which reached 95% accuracy at $143. Despite having fewer parameters (774M *vs.* 1.5B), GPT-2 Large delivered better performance at a fraction of the cost, making it the more cost-effective choice. Ultimately, the task here is so specific that it does not require all of the capabilities of larger LLMs.

For the zero-shot use case, we benchmarked the same system with GPT-4o (gpt-4o-2024–08-06), OpenAI's most recently released model at the time of publication. Despite GPT-4o’s broader generalization capabilities due to its larger parameter size and extensive pre-training, it underperformed compared to our fine-tuned GPT-2 models on familiar terminology. GPT-4o achieved an average of 25% top-5 accuracy (±5%) across all tests, modestly improving on our models’ 17% OOD accuracy, but falling far short of our models’ ≥90% accuracy on familiar ID terms. GPT-4o’s slight improvement in handling synonyms of standards unfamiliar to our models reflects its broader exposure to varied linguistic contexts, but it lacks the fine-tuned specialization required for high accuracy on known standards. Our results suggest that while large, general-purpose models such as GPT-4o can contribute to improved performance on novel terms, they are not sufficient alone to solve the harmonization task. Domain-specific, adaptive models remain essential for tasks such as cancer-related metadata standardization, where familiarity with the field’s specific nuances significantly boosts performance.

### 3.3 Harmonization of terms where training sets do not exist

**Figure 3.**
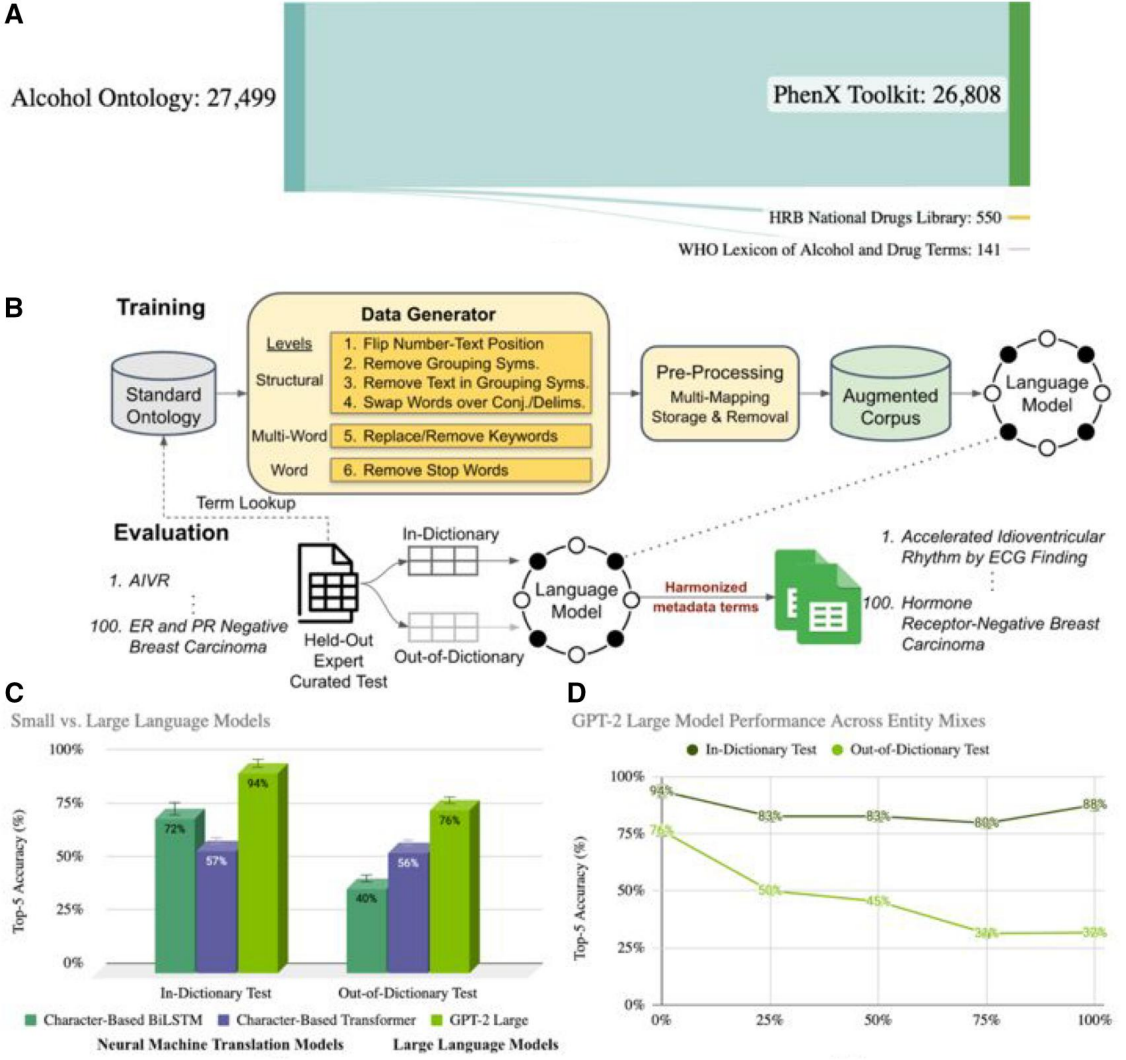
(A) The distribution of terms across standard sources in the combined alcohol vocabulary. The alcohol dataset includes 27 499 standard terms, mainly from the PhenX Toolkit. (B) Language model training and evaluation process for metadata harmonization. Standard terms flow through data generation and pre-processing to create an augmented corpus, which is used to train language models. Models are then evaluated on in-dictionary and out-of-dictionary tests. (C) Top-5 accuracy comparison of small *vs*. large language models on alcohol terminology harmonization, showing in-dictionary and out-of-dictionary test results for NMT models (Character-Based BiLSTM and Transformer) and LLMs (GPT-2 Large). (D) GPT-2 Large model performance on alcohol terms, with training conducted on varying mixes of alcohol and bacteria terminologies. ID and OOD test accuracies decline as the proportion of bacteria terms (Entity 2) in the training mix increases from 0% to 100%.

While cancer research benefits from well-established vocabularies with curated malformations, or synonyms, of standard terms, metadata harmonization is more difficult in alcohol addiction, where only standard terms exist and no curated synonym sets are available. Given our experience with alcohol research, we applied our technology to this domain. We compiled an alcohol vocabulary of 27 499 standards from authoritative sources, including the World Health Organization (WHO) Lexicon of Alcohol and Drug Terms (141) (Lexicon of Alcohol and Drug Terms), the Health Research Board (HRB) National Drugs Library (550) (Research Glossary), and the consensus measures for Phenotypes and eXposures (PhenX) Toolkit (26 808) (Hamilton *et al.* 2011) (Fig. 3A). Unlike the cancer vocabulary, which includes multiple synonyms per standard, this set contains only standardized terms, creating a challenge for training harmonization models in alcohol and other specialized fields with similar limitations.

With only the standards available, we generated synthetic synonym variations of the standard terms (Fig. 3B), which served as the training corpus for model development. Six transformations of three distinct types were applied to the standards (see Section 2), and the pre-processing module handled multimapping instances and removed conflicting variations as discussed for the cancer vocabulary. This produced a training corpus of over 200K synonym-to-standard mappings.

We trained two types of language models, Small Neural Machine Translation (NMT) models and LLMs, on the augmented terminology corpus and evaluated their harmonization performance (Fig. 3C). The Character-Based BiLSTM achieved 72% ID and 40% OOD accuracy and processed terms in under 0.1 seconds each, while the Character-Based Transformer reached 57% ID and 56% OOD with a similar speed. GPT-2 Large achieved 94% ID and 76% OOD accuracy but required 1.4 seconds per input (300 tokens/second) and about 8 hours to fine-tune, compared to 2 hours for the Bi-LSTM and 6 hours for the Transformer. These results highlight the complementary strengths of small, specialized models that offer high throughput and larger models that generalize better to unseen terms.

Finally, to further explore model generalizability and domain adaptation capabilities, as we did with different semantic types in cancer, we expanded our investigation to microbiology, specifically bacteria names, building on our past performance (Yang *et al.* 2022, Eslami *et al.* 2023) and the rising concern of antimicrobial-resistant (AMR) bacteria. Given the growth in funding to study AMR bacteria, with over $8 billion invested globally between 2008 and 2030 (Wasan *et al.* 2023), we anticipate the generation of hundreds of thousands of datasets, each contributing additional taxonomic representations to the research landscape. Therefore, we curated 627 937 standard names of bacteria drawn mainly from the National Center for Biotechnology Information (NCBI) Taxonomy (627 793) (Schoch *et al.* 2020) with contributions from our prior work (Yang *et al.* 2022) with National Institute of Standards and Technology (NIST) (144). Both ontologies share the characteristic of containing only standard terms, setting the stage for investigating harmonization techniques in fields where synonym diversity is limited and exploring the potential for cross-domain harmonization. Next, an entity mixing experiment was conducted by blending alcohol and bacteria terminology (Fig. 3D). As the proportion of bacteria terms (Entity 2) increased from 0% to 100%, a steady decline in the GPT-2 Medium model’s performance was observed in both ID and OOD tests. ID accuracy decreased from 92% to 65%, while OOD accuracy dropped from 51% to 13%, highlighting the difficulty of maintaining accuracy across diverse domains even with synthetically generated data and suggesting that domain-specific models may be required for optimal performance.

## 4 Discussion

An equivalent of a “drag and drop” curation process for biomedical research will significantly streamline the application of AI in the domain. We showed that language models can be used in a variety of ways (fine-tuning *vs.* zero-shot) to achieve semiautomated curation. Traditional heuristic methods, such as fuzzy matching, perform well for in-dictionary terms but struggle with out-of-dictionary variations, limiting their effectiveness in real-world scenarios. Small NMT models, such as character-based BiLSTMs, offer improvements over heuristics, demonstrating non-zero OOD performance. These models excel in specialized tasks within a narrow context, but lack the broad knowledge necessary for adapting to novel terms or concepts. Medium- to large-sized models, exemplified by GPT-2, strike a balance between specificity and generalization, offering improved OOD performance while maintaining high ID accuracy. At the far end of the spectrum, very large language models, such as GPT-4o, provide superior generalization capabilities, addressing a wider range of harmonization tasks across multiple domains. However, these models come with increased computational costs and potential overgeneralization, which may introduce errors in highly specialized fields. For instance, large language models benefit from a general understanding of English, which is particularly advantageous in domains such as cancer and alcohol research, where many terms overlap with everyday language. This pre-existing knowledge base contributes to their strong performance in harmonizing representations of non-domain specific words. However, in fields such as microbiology, where bacterial names are less common in general discourse, the advantage of larger models is less pronounced.

Throughout our work, particularly with the augmentation strategies, we noticed that a training corpus of word transformations that align with the evaluation data is key to an accurate model. Beyond having a model that was rich in standards, the variations that aligned were central to achieving high standardization accuracy. This means that while data augmentation can significantly expand a training corpus through means such as extensive typo generation, this would produce an unrealistic model when deployed on real variations. This implies that even with limited initial data, creative approaches to corpus expansion can significantly enhance model performance.

The approach we presented includes no additional context beyond the variation and standard to the model. This design choice reflects the practical constraints of real-world research environments, where time and resources for data annotation are limited. However, the performance gap between ID and OOD accuracy across all model sizes suggests that additional context could improve harmonization outcomes, particularly for ambiguous terms or cross-domain applications. Future research should explore adaptive systems that enhance accuracy while maintaining contextual efficiency. These systems could employ confidence thresholds to identify cases where the model’s certainty is low, prompting a request for more contextual information from the user. This iterative approach should leverage additional context only when needed to dynamically adapt to each harmonization task’s complexity, balancing accuracy and efficiency.

Finally, the choice of model size and architecture depends on the specific requirements of the harmonization task, including the breadth of the domain, the availability of training data, and the desired balance between accuracy and generalization. While very large language models (>1 billion parameters) offer a wide range of capabilities, including image recognition and code generation, the specific task of terminology harmonization may not require this full spectrum of abilities. It is more cost-effective and practical to use a tool suited for the specific task at hand. For domains with stable, well-defined vocabularies, smaller specialized LLMs offer the best balance of accuracy and efficiency. In contrast, fields with rapidly evolving terminology or frequent encounters with novel terms benefit from larger models’ generalization capabilities, despite their slower inference speeds and higher resource demands.

## Supplementary Material

vbaf241_Supplementary_Data

## Data Availability

All datasets used in this study, including training, validation, and test splits, are available via the Netrias Hugging Face organization. This includes datasets for the cancer and alcohol-bacteria mix domains used to develop and evaluate harmonization models. Experiment results are provided in the Supplementary Materials. We also share one representative GPT-2 Large cancer model and five GPT-2 Large models trained on different alcohol-bacteria domain mixtures: (100/0, 75/25, 50/50, 25/75, 0/100). All resources are released under the Apache 2.0 license to support reproducibility and reuse.
